# Adverse reactions of targeted therapy in cancer patients: a retrospective study of hospital medical data in China

**DOI:** 10.1186/s12885-021-07946-x

**Published:** 2021-02-28

**Authors:** Ruofei Du, Xin Wang, Lixia Ma, Leon M. Larcher, Han Tang, Huiyue Zhou, Changying Chen, Tao Wang

**Affiliations:** 1 The College of Nursing and Health of Zhengzhou University, Zhengzhou, 450001 China; 2 The First Affiliated Hospital of Zhengzhou University, Zhengzhou, 450052 China; 3 School of Medical Sciences, Zhengzhou University, Zhengzhou, 450001 China; 4 School of Statistics, Henan University of Economics and Law, Zhengzhou, 450046 China; 5 Centre for Comparative Genomics, Murdoch University, Perth, WA 6150 Australia; 6 Hebi People’s Hospital, Hebi, 458030 China

**Keywords:** Adverse reactions, Cancer patients, Hospitalization, Targeted therapy, Quality of life

## Abstract

**Background:**

The adverse reactions (ADRs) of targeted therapy were closely associated with treatment response, clinical outcome, quality of life (QoL) of patients with cancer. However, few studies presented the correlation between ADRs of targeted therapy and treatment effects among cancer patients. This study was to explore the characteristics of ADRs with targeted therapy and the prognosis of cancer patients based on the clinical data.

**Methods:**

A retrospective secondary data analysis was conducted within an ADR data set including 2703 patients with targeted therapy from three Henan medical centers of China between January 2018 and December 2019. The significance was evaluated with chi-square test between groups with or without ADRs. Univariate and multivariate logistic regression with backward stepwise method were applied to assess the difference of pathological characteristics in patients with cancer. Using the univariate Cox regression method, the actuarial probability of overall survival was performed to compare the clinical outcomes between these two groups.

**Results:**

A total of 485 patients were enrolled in this study. Of all patients, 61.0% (*n* = 296) occurred ADRs including skin damage, fatigue, mucosal damage, hypertension and gastrointestinal discomfort as the top 5 complications during the target therapy. And 62.1% of ADRs were mild to moderate, more than half of the ADRs occurred within one month, 68.6% ADRs lasted more than one month. Older patients (*P* = 0.022) and patients with lower education level (*P* = 0.036), more than 2 comorbidities (*P* = 0.021), longer medication time (*P* = 0.022), drug combination (*P* = 0.033) and intravenous administration (*P* = 0.019) were more likely to have ADRs. Those with ADRs were more likely to stop taking (*P* = 0.000), change (*P* = 0.000), adjust (*P* = 0.000), or not take the medicine on time (*P* = 0.000). The number of patients with recurrence (*P* = 0.000) and metastasis (*P* = 0.006) were statistically significant difference between ADRs and non-ADRs group. And the patients were significantly poor prognosis in ADRs groups compared with non-ADRs group.

**Conclusion:**

The high incidence of ADRs would affect the treatment and prognosis of patients with cancer. We should pay more attention to these ADRs and develop effective management strategies.

## Background

According to the latest global cancer data report, there are approximately 18.1 million new cancer cases in the world in 2018 [1]. In China, a total of 3.299 million people had been diagnosed as cancer in 2015, with an average of more than 10 thousand people per day. Thus, cancer has emerged as a worldwide health problem [2]. A study showed the 5-year overall survival rate of patients with advanced cancer is only 2% ~ 27% following traditional treatment methods such as chemotherapy and radiotherapy [3]. It is difficult to further improve the treatment efficacy of traditional treatment due to the limitations caused by toxicity and ADR [4]. Precision medicine fully considers the individual variability and differences among patients’ treatment strategies. With the development of bioinformatics technology, we could classify some cancers based on their specific molecular expressions. These advances have further allowed the identification of molecular therapeutic targets specific to cancer cells, thus providing a framework whereby therapies can be specifically matched to corresponding molecular targets [5]. By targeting the complex network of signaling pathways that regulates cell proliferation, angiogenesis, and apoptosis (cell death), researchers have developed new targeted agents that interfere with the growth and proliferation of cancer cells [6].

Several studies showed that the targeted therapy could improve the overall survival (OS), progression-free survival (PFS), and response rate (RR) of cancer patients [7, 8]. Moreover, compared with chemotherapy, targeted therapy has lower toxicity, better tolerance and reduced hospitalization time [9]. Although the lethal ADR is lower than chemotherapy, targeted drugs need to be used for extended periods or even indefinitely, which produce the high incidence (80%) for some ADRs in the process of targeted therapy [10]. The main characteristics of the ADR from targeted therapies are as follows: 1) they occurred in multiple organ systems; 2) different ADRs were within different targeted drugs (in terms of types, frequency and severity); 3) most of them occurred in the early stage and would not aggravate with the progress of treatment; 4) most of them were mild and could be controlled effectively; 5) Some of them were intolerable to patients required for discontinuation or other interventions. Therefore, it is very important to control the ADRs to achieve optimal clinical medication and benefit the prognosis of patients with cancer [11].

According to a recent study, the incidence of ADRs dominated in skin and mucous were as high as 86.4% during the targeted therapy [12]. Other ARDs include gastrointestinal reactions, hypertension, coagulation disorders and cardiotoxicity, which produced poor compliance correlated with the negative impact on the QoL and daily activity of patients with cancers [13]. Some studies showed that about 32% of patients with targeted molecular treatment had to discontinued the plan due to ADRs, which led to poor prognosis including cancer recurrence or progression [14]. Overall, it is imperative to focus on the ADRs of targeted therapies to improve the treatment response and the QoL. To this end, we should not only deal with the common ADRs but also be alert to the difficult ones [15]. However, there are few studies associated with ADRs of targeted cancer therapy now. And most of the published studies did not investigate other characteristics such as time and duration of ADRs of the treated cancer therapy-related ADRs [16]. Moreover, few studies explored the correlation between ADRs and prognosis of patients with cancers, and on the situation of targeted drug treatment [17].

**Fig. 1:**
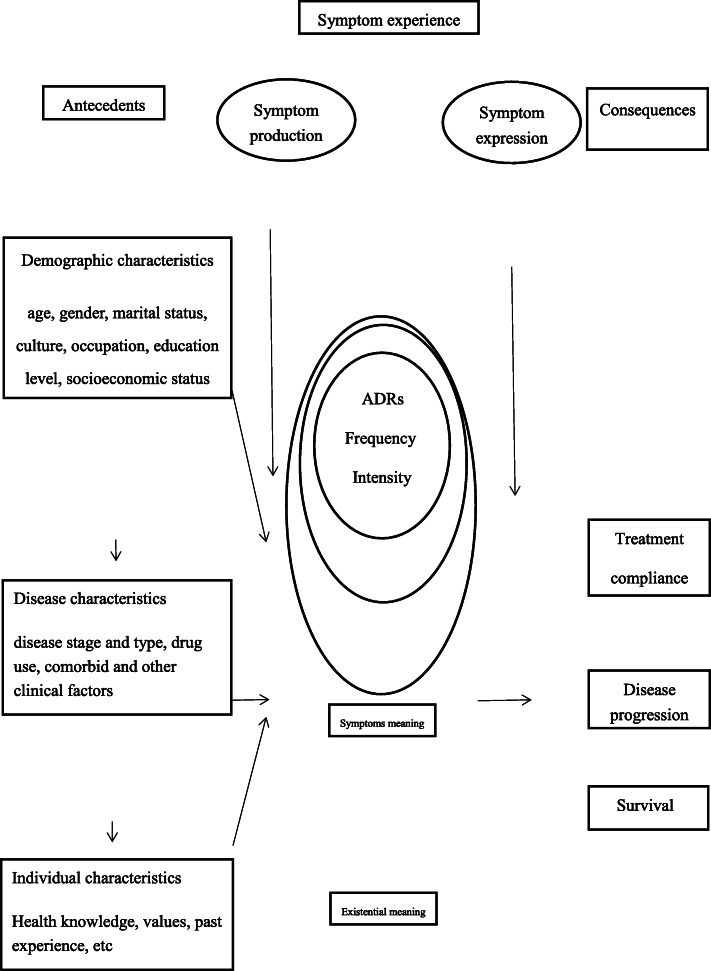
Framework of research

To provide effective identification and evaluation for ADRs, this study which guided by the theory of symptom experience model (SEM) [18] was designed to describe the incidence and characteristics of ADRs in cancer patients with targeted therapies and to investigate clinical outcomes associated with ADRs based on clinical data retrospectively. The SEM provide a complete definition of symptom experience, including antecedents, symptom experience, process and result. The main content of the model is symptom, and the symptom experience has four aspects, including the perception of symptom frequency, intensity, perplexity and symptom meaning; the influencing factors (antecedents) contain demographic characteristics, disease characteristics and individual characteristics. Outcome indicators are the results of symptom experience, including adjustment to illness, QoL, mode, functional status, disease progression and survival. And then we chose some variables from 3 modules of demographic characteristics, disease characteristics and individual characteristics in this theory and explore whether these variables are influencing factors of ADRs. Meanwhile, we described the symptom experience of ADRs caused by targeted therapy including ADRs intensity, frequency and meaning. Finally, we chose outcome indicators according to the consequences of SEM (Fig. 1).

We aim to describe the actual situation and ADR of targeted therapies in cancer patients. We also aim to understand the impact of ADR on patients, then to provide basis for clinical workers to effectively identify and accurately evaluate ADR and carry out targeted intervention measures.

## Methods

### Study design, setting and samples

A retrospective cohort design was selected for analysis with prospective data collected from electronic medical records (EMRs) between Jan 2108 and Dec 2019 by three nurses in the oncology department of three hospitals in Henan, China. The samples were non-probabilistic selection. Patients’ records were included in the analysis if they (1) were diagnosed with cancer between January 2017 and December 2017; (2) were cancer patients with targeted therapy and (3) were more than 18 years old. The informed consent was not required by using the de-identified and administrative data. The sample size was determined with the time, setting and type of patients reviewed. The sample frame was refined to include 1897 clients.

### Population selection and data variables

Medical and nursing records were excluded if they were incomplete or without complete ADRs records. ADRs are harmful and irrelevant reactions when normal dose drugs are used to prevent, diagnose, treat diseases or regulate physiological functions [6]. According to intensity, ADR can be divided into three levels of mild (ADR that the subject tolerates well, causes minimal discomfort and does not interfere with daily activities), moderate (ADR that are bothersome enough to interfere with the normal execution of daily activities) and severe (ADR that do not allow daily activities) [19]. We identified these ADRs caused by targeted drugs based on the events that these ADRs began to appear with the application of targeted drugs, and decreased or disappeared when these drugs were reduced or stopped. Moreover, according to the ADR / Event Association Evaluation Criteria in the guidelines for the use of ADRs terms, the reports with “possibly unrelated” and “unable to be evaluated” were excluded. Finally, a total of 485 patients were analyzed and only 296 patients were with an included code for ADRs. Then we examined the following areas: patients’ socio-demographic characteristics (age, gender, education level, place of residence, family income, medical insurance); disease characteristics (primary cancer, disease stage and type, drug use, comorbidities: calculated as the number of chronic diseases included in the Charlson Comorbidity Index (CCI)), ADRs (type, intensity, start time, duration); treatment compliance (discontinuing medication, change medication, dose adjustment, take medicine on time); disease progression (recurrence, metastasis) and overall survival.

### Data collection

The de-identified EMRs data was collected by nursing staffs working in the oncology departments in three hospitals and three graduate nursing students in Zhengzhou university. One student extensively involved in the ADRs study trained the other two students to extract data. Interrater reliability was 98% ~ 100% between the trainer and the other two students. The data, collected from medical and nursing records and consent documents, was coded anonymously according to Preferred terms (PT) of Medical Dictionary for Regulatory Activities (MedDRA)) [20]. The patients were divided into ADR group and none-ADR group according to whether they had an ADR or not.

### Statistical analysis

Data were entered into the SPSS version 21 (IBM, Armonk, NY, USA) for descriptive analysis. Continuous data were presented as mean and standard deviations or as medians and interquartile ranges, categorical variables were shown as proportions with 95% confidence intervals. In this matched study, the descriptive statistics for the two broad groups were displayed. The statistical significance of difference between two groups was evaluated with chi-square test. And univariate and multivariate logistic regression with backward stepwise selection were performed to examine any differences in the characteristics of cancer patients with undergoing targeted therapy using a dichotomous outcome representing patients with ADRs or not. Logistic regression models were developed with the dependent variable of ADRs. The univariate Cox regression were selected for univariate survival analysis between two groups. The independent variables of interest were the other variables available in the EMRs.

## Results

### Sociodemographics of patients enrolled into this study

**Fig. 2:**
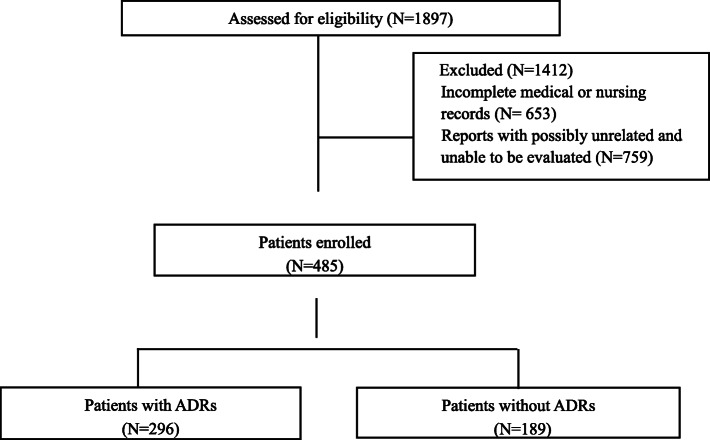
The flowchart of patient enrollment and exclusion

A total of 1897 patients were selected for this study, but 1412 patients were excluded based on the screen criteria. Finally, a total of 485 patients were enrolled into this study for further analysis (Fig. 2). The socio-demographic characteristics of these samples were shown in Table 1. About 61.0% of the patients were with ADRs. We could find that the number of patients with and without ADRs was significantly different among lung, gastric and colorectal cancer patients (*P* = 0.008, 0.007 and 0.006, respectively).

**Table 1:** Demographic and disease-related characteristics among patients with or without ADRs (univariate logistic regression)

Characteristics		Cases, *n* (%)	ADR		OR ^a^ (95%CI)	*P* value
			With, (%)	Without, *n* (%)		
All		485 (100)	296 (61.0)	189 (39.0)		
Age: Mean (SD)		63.4 (11.8)	67.8 (12.6)	59.5 (11.3)		
Age	≥60	294 (60.6)	202 (68.2)	92 (48.7)	**3.247 [1.564–7.681]**	0.014*
	<60	191 (39.4)	94 (31.8)	97 (51.3)	1	
Sex	Male	261 (53.8)	161 (54.4)	100 (52.9)	1.014 [0.386–1.714]	0.325
	Female	224 (46.2)	135 (45.6)	89 (47.1)	1	
Education Level	College and above	155 (32)	77 (26)	78 (41.3)	**0.713 [0.431–0.950]**	0.017*
	High school and below	330 (68)	219 (74)	111 (58.7)	1	
Place of residence	Urban	194 (40)	114 (38.5)	80 (42.3)	2.192 [0.689–5.237]	0.189
	Rural	291 (60)	182 (61.5)	109 (57.7)	1	
Family income (Yuan)/M	>10,000	56 (11.5)	35 (11.8)	21 (11.1)	1.885 [0.332–6.457]	0.846
	5001 ~ 10,000	227 (46.8)	133 (44.9)	94 (49.7)	0.912 [0.420–1.518]	0.092
	3001 ~ 5000	175 (36.1)	113 (38.2)	62 (32.8)	3.817 [0.774–9.220]	0.332
	<3000	27 (5.6)	15 (5.1)	12 (6.3)	1	
Primary cancer	Lung	122 (25.2)	88 (29.7)	34 (18)	**3.892 [1.521–10.109]**	0.008**
	Renal	86 (17.7)	59 (19.9)	27 (14.3)	1.732 [0.458–6.256]	0.122
	Gastric	49 (10.1)	22 (7.4)	27 (14.3)	**3.069 [1.746–9.961]**	0.007**
	Colorectal	53 (11)	23 (7.8)	30 (15.9)	**3.549 [1.472–9.135]**	0.006**
	Breast	89 (18.4)	59 (19.9)	30 (15.9)	1.776 [0.753–3.158]	0.084
	Non-Hodgkin’s Lymphoma	56 (11.5)	34 (11.6)	22 (11.6)	4.711 [0.843–7.953]	0.569
	Others	30 (6.2)	11 (3.7)	19 (10)	1	
Medical insurance	Yes	383 (79)	231 (78)	152 (80.4)	3.791 [0.654–7.824]	0.264
	No	102 (21)	65 (22)	37 (19.6)	1	
CCI	>2	240 (49.5)	178 (60.1)	62 (32.8)	**4.139 [1.418–7.027]**	0.003**
	≤2	245 (50.5)	118 (39.9)	127 (67.2)	1	
Duration of medicine	≤3 months	262 (54)	98 (33.1)	164 (62.1)	**0.647 [0.463–0.815]**	0.004**
	>3 months	223 (46)	198 (66.9)	25 (37.9)	1	
Combination of medicine	Yes	286 (59)	201 (67.9)	85 (45)	**1.736 [1.486–4.153]**	0.028*
	No	199 (41)	95 (32.1)	104 (55)	1	
Route of administration	Intravenous	209 (43.1)	164 (55.4)	45 (23.8)	**3.128 [2.066–7.550]**	0.007**
	Oral	179 (36.9)	100 (33.8)	79 (41.8)	1.806 [0.538–6.872]	0.075
	Subcutaneous	65 (13.4)	25 (8.4)	40 (21.2)	1.257 [0.296–1.836]	0.066
	Intrathecal	7 (1.4)	3 (1.0)	4 (2.1)	2.129 [0.588–4.967]	0.125
	Intraperitoneal	25 (5.2)	4 (1.4)	21 (1.1)	1	

Abbreviations: ADR, adverse reactions; CCI, Charlson Comorbidity Index

# Unmarried = single, divorced, and widow;

* *P* values are statistically significant

a Binary logistic regression models were computed for each characteristic separately and the ADR was included as an independent variable

*P<0.05, **P<0.01

### Clinical factors associated with ADRs

Multivariate logistic regression was performed to examine ADR-related factors. The univariate comparison showed that old age (OR = 1.769, *P* = 0.019), high education level (OR = 0.724, *P* = 0.017), CCI > 2 (OR = 1.715, *P* = 0.003), duration of treatment> 3 months (OR = 1.694, *P* = 0.004), combined treatment (OR = 1.488, *P* = 0.028) and route of administration (OR = 1.652, *P* = 0.034) displayed significant correlation with ADRs (Table 2). Furthermore, we could find that patients with older age, lower education level, CCI > 2, the treatment lasting more than three months, combination of medicine and intravenous administration showed significantly more ADRs. Therefore, we inferred that age, education level, comorbidities, duration of medicine, combination of medicine and route of administration were independent factors for ADRs by multivariate logistic regression.

**Table 2:** Demographic and disease-related characteristics for predicting ADRs (multivariate logistic regression)

Factors ^a^	B estimates	OR	95% CI	*P* value
Age ≥ 60	0.842	1.769	1.248–1.728	0.022*
Education level (High school and below)	−0.624	0.724	0.576–0.962	0.036*
CCI (> 2)	0.910	1.715	1.021–1.824	0.021*
Duration of medicine (> 3 months)	0.766	1.694	1.094–1.743	0.022*
Combination of medicine (yes)	0.734	1.488	1.427–1.655	0.033*
Route of administration (Intravenous)	0.689	1.652	1.468–3.935	0.019*

(multivariate logistic regression)

*Abbreviations*: *ADR* adverse reactions, *CCI* Charlson Comorbidity Index, *OR* Odds ratio, *CI* Confidence interval;

a The reference categories were age, education level, CCI, duration of medicine, combined treatment and route of administration respectively

*P<0.05

### Characteristics of ADRs in the targeted treatment

Of the patients with ADRs, a total of 646 patients had more than one kind of ADRs (Table 3). In this table, we could identify a total of 13 ADRs and discovered the top 3 ADRs as skin accessories, fatigue and mucosal damage. Meanwhile, we also found that most of ADRs appeared at the early stage of the targeted treatment (≤ 3 months, 92.4%), and most of the duration of ADRs was less than 3 months (71.8%). For the ADRs grading, the dominated patients were with less than Grade III (84.2%). Generally, we could figure out the overall characteristics of ADRs from this analysis, which was important for clinical prevention.

**Table 3:** Characteristics of ADRs

Characteristics	Frequency	%	Characteristics	Frequency	%
**Type of ADR**			**ADR grading**		
Skin and its accessories	125	19.3	Grade I	248	38.4
Fatigue	109	16.9	Grade II	153	23.7
Mucosal damage	94	14.6	Grade III	143	22.1
HBP	76	11.8	Grade IV	70	10.8
Gastrointestinal discomfort	69	10.7	Grade V	32	5.0
Insomnia	59	9.1	**Duration**		
Hand-foot syndrome	45	7.0	<1 M	31.4	
Cardiotoxicity	24	3.7	1 M~	40.4	
Hematological system disorders	23	3.6	3 M~	21.2	
Thrombus	8	1.2	6 M~	7.0	
Interstitial pneumonia	4	0.6			
Others	10	1.5			
**The start time of ADR**					
<1 M	333	51.5			
1 M~	264	40.9			
3 M~	49	7.6			

### Treatment compliance correlated with ADRs

In order to investigate the compliance of patients with targeted treatment, we analyzed 5 clinical events (discontinuing medication, change medication, adjust drug dosage, not taking the medication on time) between two groups with or without ADRs. As a result, we discovered that patients with cancer were more likely to occur worse treatment compliance in the ADR group than those in non-ADR group (all *P* < 0.05, Table 4). The number of patients of discontinuing medication, changing medication, adjust drug dosage, not taking the medication on time were statistically significant difference between ADR and non-ADR group. And 33% (98/296), 53% (157/296) and 62.8% (186/296) of patients in ADR group changed, adjusted and did not taking the medication on time, respectively.

**Table 4:** The difference of treatment compliance between ADR and non-ADR group

Variables/group	ADR group (n = 296)%	Non-ADR group (*n* = 189)%	χ2	*P* value
Discontinuing medication	184 (62.2)	68 (36)	31.68	0.000
Change medication	49 (16.6)	11 (5.8)	12.26	0.000
Adjust drug dosage	157 (53)	27 (14.3)	73.58	0.000
Not taking the medication on time	110 (37.2)	36 (19)	17.99	0.000

**Fig. 3:**
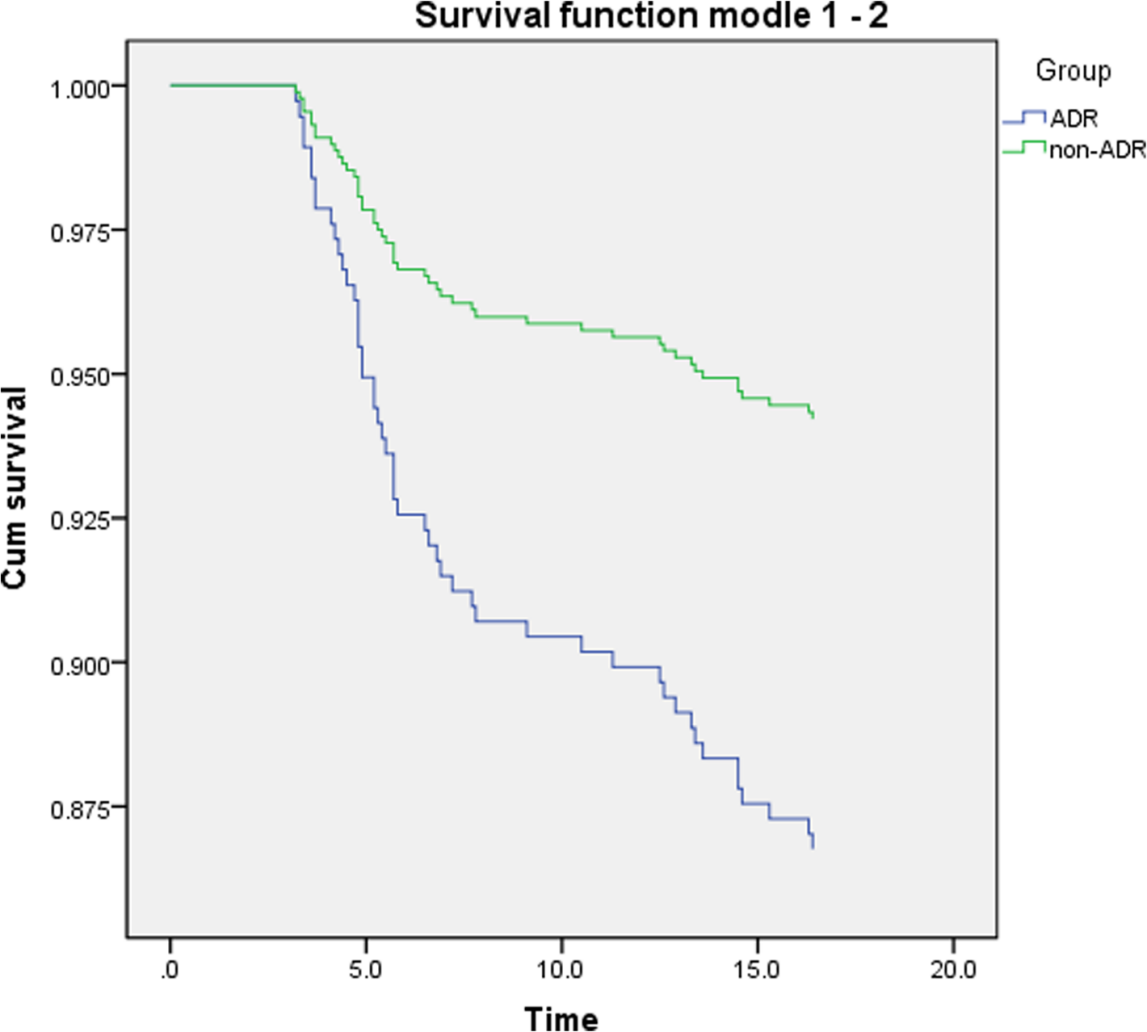
Recurrence survival curve between patients with and without ADRs

**Fig. 4:**
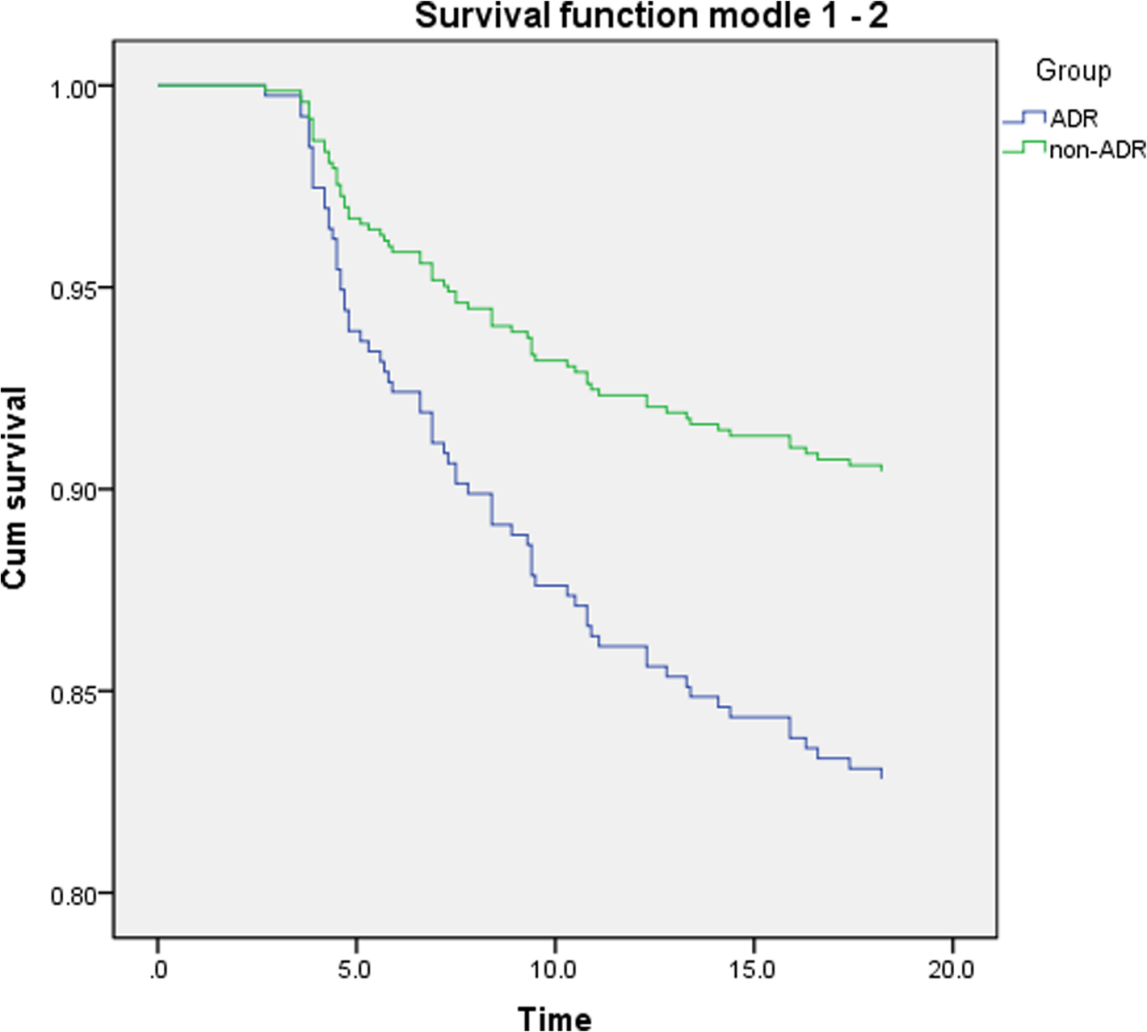
Metastasis survival curve between patients with and without ADRs

**Fig. 5:**
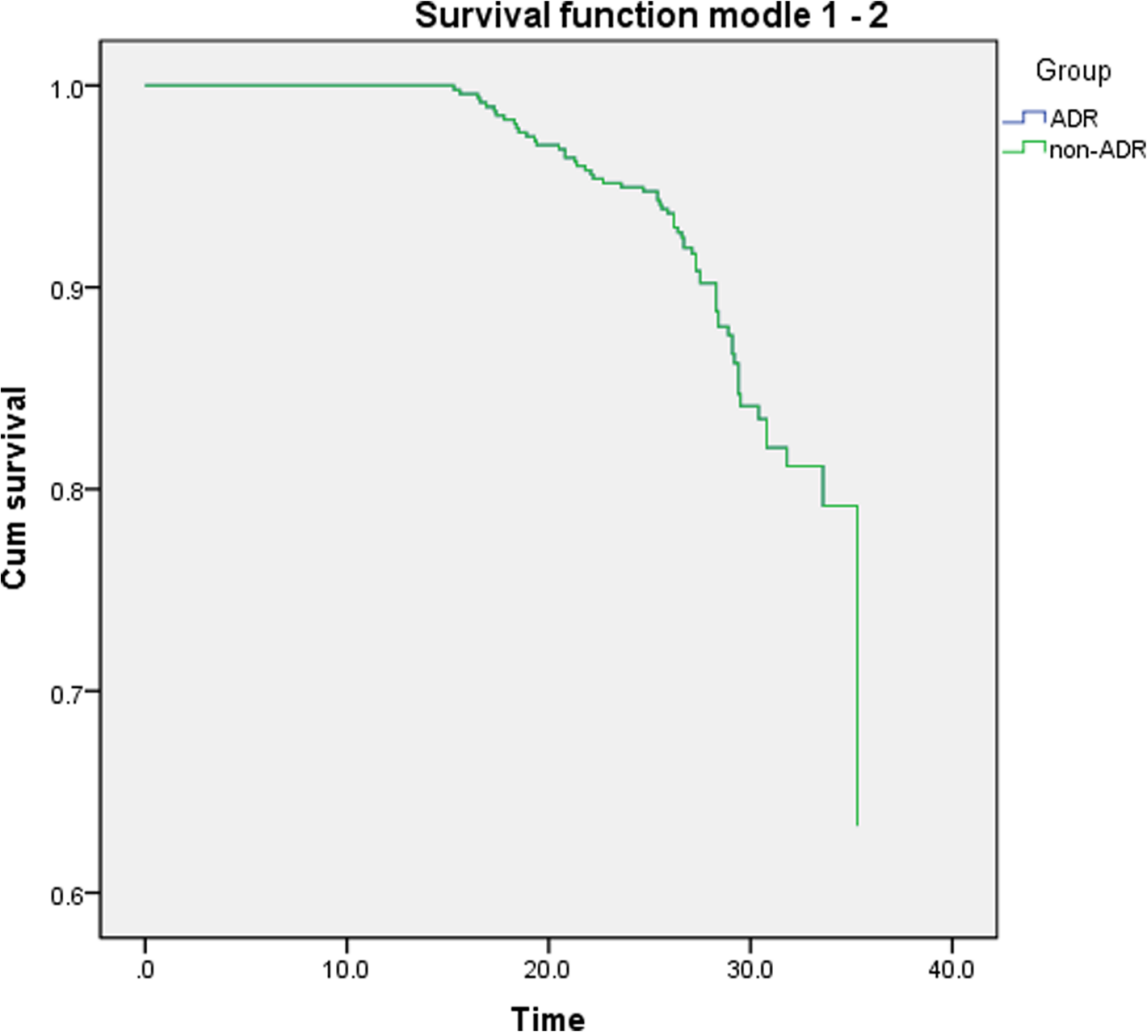
Overall survival analysis between patients with and without ADRs

Furthermore, we performed univariate Cox regression to compare the different probability of recurrence, metastasis and overall survival between the two groups with and without ADRs (Figs. 3, 4, 5). The result showed that the number of patients of recurrence (*P* = 0.000) and metastasis (*P* = 0.006) were statistically significant difference between ADR and non-ADR group. Compared with patients with ADRs, the risk of recurrence (HR = 0.539, 95%CI: 0.393–0.739) and metastasis (HR = 0.650, 95%CI: 0.478–0.885) in patients without ADRs was lower than that in patients with ADRs. However, there was no statistical significance for overall survival between these two groups (*P* = 0.365, Table 5). All the results above suggested that cancer patients with ADRs would occur treatment compliance and produce poor prognosis.

**Table 5:** Comparison of disease progress and overall survival according to ADR group

Items	χ^2^	*P* value
Recurrence	14.798	0.000
Metastasis	5.512	0.006
Death	0.820	0.365

## Discussion

According to this study, patients with ADRs had different socio-demographic characteristics, treatment compliance, disease progression and overall survival compared to those without ADRs. Our study expanded on previous studies by providing a comprehensive evaluation on pathological characteristics related to ADRs in patients with targeted therapies. To our knowledge, few studies had examined the features of ADRs of targeted cancer therapies [21]. Previous studies only investigated the type and intensity of ADRs but the occurrence time and duration of ADRs [13, 22]. In addition, no study reported outcomes related to ADRs and impacts on patients in China. Through this research, we discovered some clinical characteristics such as age, education level and comorbidities and some treatment strategies including combinatorial treatment and administration route were associated with ADRs. Meanwhile, we summarized more comprehensive characteristics of ADRs, including the types, grading, occurrence time and duration, which made up for the incomplete evaluation and analysis of ADRs. Patients with ADRs had poor treatment compliance, and experienced a higher rate of discontinuation, change and adjustment of medication, and tended to not taking medicine on time. Moreover, patients with ADRs had higher rates of cancer recurrence and metastasis.

In this study, 61% patients with a targeted therapy occurred ADRs, which was lower than previous observations [23, 24]. In fact, the proportion of patients with ADRs might be far more than 61%. This is because that in this study, we excluded patients with incomplete ADRs information in their medical records, and actually, there were patients who had ADRs, but their ADRs information was not recorded regularly. We observed that there were differences in the number of patients with or without ADRs in some cancer types, however, this result had no practical significance. Because patients took in different drugs, the characteristics of ADRs were not the same. On the other side, as for patients with different types of cancer had different conditions, complications and prognosis which might impact on the ADRs. This study also showed that age and comorbidities were positive influence on ADRs, and older patients and patients with more comorbidities were more likely to produce ADRs which was consistent with the conclusion from Tristan and Daud et al. [4, 25]. It might be correlation with the decrease of drug metabolism or the damage of liver and kidney function in the elderly. Additionally, comorbidities led patients to suffer from complicated conditions, which might also stem from using a number of medications correlated with the ADRs caused by drug interaction. Furthermore, the interaction between diseases might produce worse physical, emotional and social function [26].

Especially, this study highlighted the importance of health education. We concluded that the education level of patients affected the occurrence of ADRs. When the underlying reasons were investigated, it was found that patients with low education level could not understand the targeted therapies, and therefore could not accept the health information related to drug use smoothly. This research showed that more than 90% of patients undergoing targeted therapy, which indicated that it was necessary to know drug-related knowledge and to solve ADRs through health education by medical staffs [27].

Therefore, medical staffs should pay more attention to elderly patients or patients with more comorbidities. We should know and monitor the disease changes, treatment strategies, drug efficacy and medication of elderly and comorbid patients. What’s more, it is very important to strengthen early assessment and risk management, which are of great significance to improve drug response and reduce ADRs. At the same time, we also suggest guiding the elderly and comorbid patients to carry out self-management, and to remind and supervise patients to implement ADRs management. By the effective health education, we could improve the understanding of targeted therapies and the ADRs of these patients, allowing them the ability to become familiar with the drugs they used [28]. We should also teach patients to record utilizing medication diaries and assist patients into managing comorbidities, in order to balance the application between other drugs and targeted drugs. Moreover, we should try to prevent the ADRs of targeted therapies in advance, visit patients before targeted treatment to evaluate and review patients, then discuss with patients to develop educational goals and plans to facilitate ADRs prevention.

Combined treatment and route of drug administration may affect the ADR of targeted therapies, which has been confirmed by Staats, Jui-Chun, and Bhullar et al., and is consistent with our research [6, 29, 30]. As reported previously by Muro [21], this study indicated that the duration of drug administration didn’t affect the ADRs of targeted therapy. The ADRs of drugs were mostly caused by the components of the drugs themselves. Although the toxicity of these drugs didn’t change over time, some toxicity appeared earlier while some later. After intravenous administration, drugs rapidly distributed to the whole body. Drugs without metabolism and detoxification could not only kill tumor cells but also damage normal tissues and organs [31], thus causing ADRs in multiple systems / organs. When various drugs were combined in the clinical treatment, the physicochemical properties, drug reactions, metabolism and excretion may interfere with each other. At the same time, the accumulation of toxic components in the body increased the possibility of ADRs [32]. Therefore, it is necessary to formulate reasonable drug treatment strategies, enhance the awareness of rational drug use, and closely monitor the drug administration and use.

We also discovered that the top five ADRs in this study were damage to the skin, fatigue, mucosal damage, hypertension and gastrointestinal discomfort. Most of the ADRs of targeted therapy were chronic [17], we found more than half of the ADRs occurred within 1 month. However, there were also some ADRs such as cardiotoxicity, which occurred immediately after administration. A few ADRs such as thrombosis and interstitial pneumonia may appear after 3 months of administration. For the most common ADRs of skin, 60% of the patients had skin ADRs within 1 month after treatment. Among them, acneiform eruptions appeared in the first 2 weeks of treatment, 46.5% of patients had dry skin in the first month, and paronychia was more common after 2 months of treatment. During this study, ADRs was graded according to the latest CTCAE 5.0 standard issued by the U.S. department of health and human services in 2017 [19]. We concluded that 84.2% of ADRs were mild to moderate in severity, which was consistent with the view that the degree of ADRs of targeted therapies was lower than that of chemotherapy [33]. The more severe ADRs included cardiotoxicity, coagulation dysfunction and interstitial pneumonia, these ADRs were mostly fatal. Generally, skin ADRs were mild, but severe rash can also lead to death. Another characteristic of the ADRs of targeted therapies was that the duration of ADRs was long. 68.6% ADRs of the patients in the study lasted for more than 1 month. In addition, most of the ADRs occurred repeatedly, and pulmonary fibrosis caused by interstitial pneumonia was even permanent.

As for the impact of ADRs on patients and ADRs related outcomes, we collected data on treatment, disease progression and prognosis, including drug use, recurrence, metastasis and death. We observed that patients with ADRs might change treatment programs and drugs, adjust drug dosage and even discontinue treatment. Meanwhile, compared with patients without ADRs, patients in ADRs group with orally administered drugs stopped taking medication and reduced drug dosage more due to being unable to tolerate the ADRs. The compliance of patients with orally targeted drugs decreased, while the incidence of missed dosages and not taking drugs on time increased, this was confirmed by Sano et al. [34]. Therefore, it is important to manage ADRs well. In this study, 62.2% of patients stopped taking drugs and 62.8% did not take drugs on time due to ADRs. ADRs resulted in negative impact on the QoL, physical function, daily activity, social and emotional function of cancer patients, which reduced the desire of patients for treatment, affected their work and study, so as to make patients resist taking medicine [35]. What’s worse, these patients suffered for a long time, resulting in the sense of helplessness and apparent social participation disorder, anxiety and psychological distress, these were all reasons why it was difficult for patients to carry out treatment smoothly.

In this study, we used survival analysis to compare the disease progression and prognosis of patients with and without ADRs. The rates of patients who had cancer recurrence or metastasis were 46.6 and 43.1%, a small proportion patients died (13%). We can see, within the time frame of this review, the proportion of endpoint events was less than 50%, so we used Cox regression rather than K-M method for analysis. The results showed recurrence or metastasis occurred more in patients with ADRs. Due to the influence of ADRs on physiology and psychology, patients’ health condition was worse which reduce the tolerance of patients to resist disease. Moreover, the normal progress of treatment is hindered, and because of the poor compliance, the drugs did not achieve the desired effect so that the ability of drugs to control and treat the disease was declined too, which led to the recurrence and metastasis of cancer [36]. Therefore, it is very important to implement ADRs management to reduce and alleviate the ADRs in order to guarantee the treatment process and improve the medication compliance to prevent patients from stopping, reducing or not taking medicine on time.

### Advantages and limitations

Our study supplemented the situation in mainland China about ADRs of patients who received targeted therapies. Previous studies only analyzed the type and classification of ADRs, but we comprehensively interpreted the characteristics of ADRs, further discussed the start time and duration of ADRs, and explored some influencing factors of ADRs. Moreover, our research was based on SEM theory, which provided the basis for determining the research program and the selection of variables in the research. However, it is necessary to highlight some limitations. A lot of ADRs information in the EMRs reviewed in this study were missing or incomplete, which indicated that sometimes, there was no reporting or recording of ADRs in clinical practice, or there was no standard ADRs records, which leaded to difficulties in extracting information and means that the results could be biased. The reasons may be that most of the ADRs were chronic and not serious, so, medical staffs were not aware of the importance. It should be noted that we had reviewed only the last 2 years so that data collection was limited. However, there was no difference in the outcome of death between ADR and non-ADR group, it might require a longer duration to review. Similarly, although we found differences in recurrence and metastasis outcomes between the two groups, a longer-term review could be more meaningful. At the same time, the impact of ADRs on patients and the prognosis of the disease also includes the quality of life, functional status, psychological status, OS and PFS of patients, more prospective research to explore these indicators are of great significance to clinical practice and theory.

### Implication

This study provided data on ADRs of cancer patients with targeted treatment, and analyzed the influencing factors and outcomes of ADRs, which indicated important information for medical staff, allowing them to pay more attention to ADRs of targeted treatment of cancer patients. Meanwhile, the results of this study promoted the identification, monitoring, evaluation and recording of ADRs and provided ideas and premise for intervention research of ADRs. Furthermore, we supply a reference for clinical practice, in order to help and improve clinical decision-making. Additionally, the results of this study were of great significance to promote the safety of patients, and provided the basis for further understanding the ADRs of targeted therapies and the factors that should be paid attention to during medication administration and use. On the other side, health-care providers should pay attention to the factors identified in this study and consider the following strategies: 1. Clinical strategies: principles of coping with ADRs: before treatment: ① Baseline comorbidity assessment and intervention (1) Comprehensively evaluating whether the patient has some risk factors, balancing the advantages and disadvantages of treatment, and developing individualized treatment plan, (2) Controlling other comorbidities, (3) Symptomatic treatment; ② Patient education (1) Patients need to fully understand the disease and treatment (education on potential ADRs), (2) Psychological education; after treatment: ① Close monitoring and treatment (1) CTCAE standard evaluation, developing standardized ADRs terms according to the specific national conditions/priorities in China in combination with the existing international standardized terminology, (2) developing specific ADRs evaluation plan, determining the key points of ADRs monitoring and identification, developing ADRs record form, the usage, dosage, course of treatment should be strictly monitored, (3) Quality of life report of patients, (4) providing fast and effective care in order to minimize the risk and severity of ADRs; ② Rapid ADRs management (1) Standard medical intervention, (2) Considering dose adjustment or interruption if necessary, (3) Actively coping with complex ADRs in multidisciplinary team mode, (4) Clinicians and patients should weigh the potential survival benefits when planning to change drugs; ③ Making reasonable follow-up strategy: (1) Following up on time, recording ADRs in detail, (2) Dynamic comparison of ADRs, finding problems and taking measures in time. 2. Prevention of common ADRs ① Skin ADRs: using skin care preparations, avoiding skin injury behaviors, skin preventive medication; ② Fatigue: ensuring adequate sleep, developing individualized exercise program, adhering to the reasonable intensity of exercise; ③ Mucositis: keeping oral hygiene and avoiding irritating diet; ④ HBP: blood pressure monitoring, controlling blood pressure below 140 / 90 mmHg, drug control; ⑤Gastrointestinal reactions: rational diet and preventive medication. 3. Measures should be taken for ADR related factors: ① We should pay more attention to the patients with older age, low education level and comorbidity, improve the physical condition of these patients in order to strengthen tolerance; ② We should formulate rational treatment strategies, closely monitor the drug use and avoid unreasonable/dangerous combinations of drugs; ③The implementation of individualized and effective health education to improve patients’ cognition to promote self-management. 4. Prevention and self-management education for patients and their families①Health education includes disease knowledge, drug knowledge and ADRs knowledge; ② Behavior guidance includes medication compliance guidance, nutrition guidance and medication guidance; ③ Skills training includes medication records, ADR identification and monitoring, ADR prevention measures, the role and application of traditional Chinese medicine.

## Conclusion

The incidence of ADRs in cancer patients during targeted therapy was high, with different features involving various systems / organs. Most of ADRs were mild to moderate in severity, while some were lethal. These ADRs were mostly chronic and occurred after one month of administration. Some ADRs existed during the treatment or even had permanent damage. Age, education level, comorbidity and medication strategy could impact on ADRs, while ADRs could also affect the treatment and prognosis of patients. Therefore, the characteristics, influencing factors and outcomes of ADRs obtained in this study could accumulate experience for clinical staff to carry out ADRs management, to improve the treatment effectiveness and ensure the safety of patients.

## Data Availability

The data generated during and/or analyzed during the current study are not publicly available, but are available from the corresponding author who was an organizer of the study.

## Abbreviations

ADR: Adverse reactions
QoL: Quality of life
OS: Overall survival
PFS: Progression-free survival
RR: Response rate
SEM: Symptom experience model
EMRs: Electronic medical records
CCI: Charlson Comorbidity Index

## References

[1] Bray F, Ferlay J, Soerjomataram I, Siegel RL, Lindsey S (2018). Global cancer statistics 2018: GLOBOCAN estimates of incidence and mortality worldwide for 36 cancers in 185 countries. CA Cancer J Clin.

[2] Yang D, Yang L, Bai C, Wang X, Powell CA (2020). Epidemiology of lung cancer and lung cancer screening programs in China and the United States. Cancer Lett.

[3] Estève J, Kricker F, Jacques MD (2015). Facts and figures of Cancer in the European Community. Can J Neurol Sci.

[4] Barnes TA, Amir E, Templeton AJ, Gomez-Garcia S, Navarro B, Seruga B (2017). Efficacy, safety, tolerability and price of newly approved drugs in solid tumors. Cancer Treat Rev.

[5] Wilkes GM (2018). Targeted therapy attacking Cancer with molecular and immunological targeted agents. Asia Pac J Oncol Nurs.

[6] Bhullar KS, Lagarón NO, Mcgowan EM, Parmar I, Jha A, Hubbard BP (2018). Kinase-targeted cancer therapies: progress, challenges and future directions. Mol Cancer.

[7] Kris MG, Johnson BE, Berry LD, Kaldal A (2014). Using multiplexed assays of oncogenic drivers in lung cancers to select targeted drugs. JAMA..

[8] Singal G, Miller PG, Agarwala V, Li G, Kaushik G, Backenroth D (2019). Association of patient characteristics and tumor genomics with clinical outcomes among patients with non–small cell lung Cancer using a Clinicogenomic database. JAMA..

[9] Schwaederle M, Zhao M, Lee JJ, Eggermont AM, Schilsky RL, Mendelsohn J (2015). Impact of precision medicine in diverse cancers: a meta-analysis of phase II clinical trials. J Clin Oncol.

[10] Tischer B, Huber R, Kraemer M, Lacouture ME (2017). Dermatologic events from EGFR inhibitors: the issue of the missing patient voice. Support Care Cancer.

[11] Porta C, Levy A, Hawkins R, Castellano D, Bellmunt J, Nathan P (2014). Impact of adverse events, treatment modifications, and dose intensity on survival among patients with advanced renal cell carcinoma treated with first-line sunitinib: a medical chart review across ten centers in five European countries. Cancer Med-Us.

[12] CLELL, HASENBANK (2017). Supportive care and Management of Treatment-Emergent Adverse Events with Targeted Therapy in non-small cell lung Cancer. J Adv Pract Oncol.

[13] Bo Z, Fang C, Deng D, Liang X (2018). Research progress on common adverse events caused by targeted therapy for colorectal cancer. Oncol Lett.

[14] Cazzaniga ME, Danesi R, Girmenia C, Invernizzi P (2019). Management of toxicities associated with targeted therapies for HR-positive metastatic breast cancer: a multidisciplinary approach is the key to success. Breast Cancer Res Tr.

[15] Dienstmann R, Braña I, Rodon J, Tabernero J (2011). Toxicity as a biomarker of efficacy of molecular targeted therapies: focus on EGFR and VEGF inhibiting anticancer drugs. Oncologist..

[16] Barton-Burke M, Ciccolini K, Mekas M, Burke S (2017). Dermatologic reactions to targeted therapy: a focus on epidermal growth factor receptor inhibitors and nursing care. Nurs Clin N Am.

[17] Lacouture M, Sibaud V (2018). Toxic side effects of targeted therapies and immunotherapies affecting the skin, Oral mucosa, hair, and nails. Am J Clin Dermatol.

[18] Terri S, Armstrong (2003). Symptoms Experience: A concept analysis. Oncol Nurs Forum.

[19] Freites-Martinez A, Santana N, Arias-Santiago S, Viera A (2020). Using the common terminology criteria for adverse events (CTCAE – version 5.0) to evaluate the severity of adverse events of anticancer therapies. Actas Dermo-Sifiliográficas.

[20] Brown DEG, Wood L, Wood S (1999). The medical dictionary for regulatory activities (MedDRA). Drug Saf.

[21] Muro YNK (2016). Challenges in molecular targeted therapy for gastric cancer considerations for efficacy and safety. Expert Opin Drug Saf.

[22] Deutsch A, Leboeuf NR, Lacouture ME, McLellan BN. Dermatologic Adverse Events of Systemic Anticancer Therapies: Cytotoxic Chemotherapy, Targeted Therapy, and Immunotherapy. Am Soc Clin Oncol Educ Book. 2020 2020-05-01; 40:485–500. 10.1200/EDBK_289911 32421446

[23] Chen CB, Wu MY, Yee NC, Lu CW, Wu J, Pei-Han K (2018). Severe cutaneous adverse reactions induced by targeted anticancer therapies and immunotherapies. Cancer Manag Res.

[24] Anna J. Lomax, Theresa Nielsen, MCaHaem N, Lydia Visintin. Clinical Nurse Consultant Support: Management of Patients With Melanoma Receiving Immunotherapy and Targeted Therapy. Clin J Oncol Nurs. 2017 2017-08-01; 21(4):E93–E98. 10.1188/17.CJON.E93-E98 28738040

[25] Daud A, Tsai K (2017). Management of Treatment-Related Adverse Events with agents targeting the MAPK pathway in patients with metastatic melanoma. Oncologist..

[26] Yamamoto K, Yano I (2018). Genetic polymorphisms associated with adverse reactions of molecular-targeted therapies in renal cell carcinoma. Med Oncol.

[27] Damiani G, Manganoni A, Cazzaniga S, Naldi L (2018). Survey of cutaneous adverse reactions to targeted cancer therapies: value of dermatological advice. G Ital Dermatol Venereol.

[28] Brunot AL, Le Roy F, Le Sourd S, Amel M Sadek, Marielle Duval, Laurence Crouzet, et al. (2018). Implementation of a nurse-driven educational program improves management. Cancer Nurs.

[29] Staats H, Cassidy C, Kelso J, Mack S (2020). Targeted molecular therapy in palliative Cancer management. Biomedicine..

[30] Chan J, Lee Y, Liu C, Shih H (2019). A correlational study of skin toxicity and quality of life in patients with advanced lung Cancer receiving targeted therapy. J Nurs Res.

[31] Patrizi A, Venturi M, Dika E, Maibach H, Tacchetti P, Brandi G (2014). Cutaneous adverse reactions linked to targeted anticancer therapies bortezomib and lenalidomide for multiple myeloma: new drugs, old side effects. J Toxicol Cutaneous Ocular Toxicol.

[32] Chan SL, Ang X, Sani LL, Hong YN, Chan A (2016). Prevalence and characteristics of adverse drug reactions at admission to hospital: a prospective observational study. Br J Clin Pharm.

[33] Gervès-Pinquié C, Daumas-Yatim F, Lalloué B, Girault A, Ferrua M, Fourcade A (2017). Impacts of a navigation program based on health information technology for patients receiving oral anticancer therapy the CAPRI randomized controlled trial. BMC Health Serv Res.

[34] Sano K, Nakadate K, Hanada K (2020). Minocycline prevents and repairs the skin disorder associated with afatinib, one of the epidermal growth factor receptor-tyrosine kinase inhibitors for non-small cell lung cancer. BMC Cancer.

[35] Holm JG, Agner T, Clausen ML, Thomsen SF (2016). Quality of life and disease severity in patients with atopic dermatitis. J Eur Acad Dermatol Venereol.

[36] Fornasier G, Taborelli M, Francescon S, Polesel J, Aliberti M, De Paoli P (2018). Targeted therapies and adverse drug reactions in oncology: the role of clinical pharmacist in pharmacovigilance. Int J Clin Pharm-Net.

